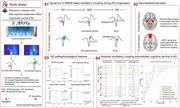# Coupled Sleep Rhythm Disruption Predicts Cognitive Decline in Alzheimer's Disease

**DOI:** 10.1002/alz70856_098741

**Published:** 2025-12-24

**Authors:** Tao Wei, Jianyang Zhou, Yi Tang

**Affiliations:** ^1^ Xuanwu Hospital, Capital Medical University, Beijing, Beijing, China; ^2^ Chinese Institute for Brain Research, Beijing, Beijing, China; ^3^ Department of Neurology & Innovation Center for Neurological Disorders, Xuanwu Hospital, Capital Medical University, National Center for Neurological Disorders, Beijing, Beijing, China

## Abstract

**Background:**

The effect of sleep on memory consolidation depends on the precise interaction of slow oscillations (SO), theta bursts, and spindles. Disruption in coupling of these sleep rhythms has been reported for individuals with Alzheimer's disease (AD). However, it is not known how the sleep rhythms evolve during AD progression and whether disrupted sleep rhythms facilitate cognitive decline in AD.

**Method:**

Here, we analyze data of 93 individuals from sleep electroencephalography (EEG), MRI, cerebrospinal fluid (CSF) AD biomarker, and two‐year cognitive assessments among three populations: AD dementia (*n* = 33), mild cognitive impairment (MCI) due to AD (*n* = 38), and cognitively normal (CN; *n* = 22).

**Result:**

Our study identifies the evolving pattern of coupled sleep rhythm disruption with advancing cognitive stages in AD. Specifically, the frequency of SO‐theta burst coupling and SO‐spindle coupling decreases from CN to MCI; the precision of SO‐theta burst coupling and SO‐spindle coupling further decline from MCI to AD dementia. The *APOE* ε4 allele and elevated amyloid and tau burden are associated with coupled sleep rhythm disruption. Hippocampal and medial prefrontal cortex atrophy are respectively linked to disruption of SO‐theta burst coupling and SO‐spindle coupling. Notably, coupled sleep rhythm disruption predicts accelerated cognitive decline over a two‐year follow‐up period. Our study presents that integrating sleep EEG with CSF and MRI biomarkers enhances the predictive ability for AD progression, which unravels the potential of sleep rhythms as monitoring and interventional targets for AD.

**Conclusion:**

Our study demonstrated that the frequency and precision of coupled sleep oscillations decreased during AD progression, and that changes in coupled sleep oscillations were associated with cognitive decline at a 2‐year follow‐up.